# A New Theory of the Nature of Water

**Published:** 1878-03

**Authors:** 


### A NEW THEORY OF THE NATURE OF WATER.

M. Maiche, in *Les Mondes*, propounds the theory, reached after numerous experiments, that water is simply hydrogen *plus* electricity, or oxygen *minus* electricity; or, in other words, that normal electrified hydrogen constitutes water, and that normal diselectiified oxygen produces the same; or that hydrogen, oxygen and water are precisely the same, differing only in degree of electrification.

